# Towards a widespread adoption of metabolic modeling tools in biopharmaceutical industry: a process systems biology engineering perspective

**DOI:** 10.1038/s41540-020-0127-y

**Published:** 2020-03-13

**Authors:** Anne Richelle, Blandine David, Didier Demaegd, Marianne Dewerchin, Romain Kinet, Angelo Morreale, Rui Portela, Quentin Zune, Moritz von Stosch

**Affiliations:** grid.425090.a GSK, Rixensart, Belgium

**Keywords:** Systems biology, Business and industry, Biotechnology

## Abstract

In biotechnology, the emergence of high-throughput technologies challenges the interpretation of large datasets. One way to identify meaningful outcomes impacting process and product attributes from large datasets is using systems biology tools such as metabolic models. However, these tools are still not fully exploited for this purpose in industrial context due to gaps in our knowledge and technical limitations. In this paper, key aspects restraining the routine implementation of these tools are highlighted in three research fields: monitoring, network science and hybrid modeling. Advances in these fields could expand the current state of systems biology applications in biopharmaceutical industry to address existing challenges in bioprocess development and improvement.

## Introduction

The emergence of high-throughput technologies is elevating the biotechnology field to the era of big data^1^. This shifting paradigm created considerable challenges for the interpretation of large datasets and the generation of meaningful outcomes impacting process and product attributes. Among the numerous computational methods developed in this context, metabolic modeling tools allowed the coherent organization of large datasets into biological networks providing nonintuitive insights on biological systems that in vivo experiments alone cannot provide^2^. These model-based approaches have been proven to be invaluable at the level of preclinical research. For example, these approaches have been used for the design of new drugs by informing target selection and for the engineering of cells by rewiring metabolism towards the production of a product of interest^3,4^.

Pharmaceutical companies are already investing substantially in computational approaches to guide drug discovery and cell design. While these important applications are out of the scope of this paper, we refer the interested readers to detailed reviews on the usage of metabolic engineering and synthetic biology in industrial context^5–8^. However, model-based methods can be used for much more than this. For instance, these methods could also be applied at the industrial level in the field of process design, monitoring and control to lower the experimental effort and increase the process robustness. Indeed, model-based methods would allow a more rational design of bioprocesses but also the objective identification of the variables to monitor and control. Therefore, the routine implementation of these methods can be expected to greatly facilitate the implementation of regulatory requirements related to the Quality by Design paradigm (i.e., systematic approach to development that begins with predefined objectives and emphasizes product and process understanding and process control) and Process Analytical Technologies initiative (mechanism to design, analyze and control manufacturing processes through the measurement of Critical Process Parameters that affect Critical Quality Attributes^9^). Numerous modeling methods exist to describe and quantify the reactions occurring in a cell under specific environments, from deterministic kinetic models to stochastic and statistical models. While kinetic modeling has been widely used for small-scale metabolic models, these mechanistic models are for the moment not scalable and suitable for genome-wide approaches due to challenges in acquiring the required kinetic parameters (i.e., rate constants, enzymes and intracellular metabolite concentrations) and computational problems associated with such complex parametric systems (i.e., model nonlinearity, parameter identifiability, and computational tractability)^10^. On the other hand, while statistical modeling is very useful for the manipulation of large complex datasets, these black-box approaches have limited capacity to generate biologically relevant information for bioprocess improvement. Therefore, the lack of clear workflows to set up specific process models based on genome-wide information have limited the potential scope of computational biology applications in industry.

However, the systems biology field is in continuous development and genome-scale metabolic networks are now available for most of the industrially relevant organisms. Therefore, we should be able to systematically predict cell metabolic behavior under different specific complex media compositions and process conditions. These types of predictions should be used to objectively determine the optimal operating conditions with respect to the desired cell phenotype and related production criteria. Unfortunately, while numerous studies have successfully used genome-scale models to rationally guide the culture process design^11–13^, their practical implementation in biopharmaceutical research and development facilities still seems in its infancy. Actually, bioprocess improvement performance is still mainly achieved by semi-empirical media and bioprocess optimization (i.e., screening different media and process conditions and analysis using statistical design-of-experiment strategies)^14^. In this paper, gaps in current knowledge and technical limitations restraining the widespread usage of metabolic modeling tools for process development (i.e., design, optimization and control) in biopharmaceutical industry are highlighted. Specifically, research topics in three fields (real-time monitoring, biological network science and hybrid modeling) are identified as key drivers for evolving the current state of some systems biology tools in industrial process engineering applications. Advances in these fields will be invaluable to address existing challenges in bioprocess development and improvement.

## Real-time bioprocess monitoring: track to better control

Real-time monitoring is a key issue for effective bioprocess operation. Indeed, one needs first to recognize and analyze cellular physiological state in function of environmental conditions to be further able to adequately manipulate operating conditions. Therefore, real-time monitoring technologies development is a critical aspect of the bioprocess automatization. Previously, only a small number of so-called “process variables” were commonly measured online in bioreactors (e.g., pH, temperature, OD, stirring). The scope of available real-time probes has now been significantly expanded thanks to the recent advances in analytical technologies and the adoption of Process Analytical Technologies initiative^15,16^.

In the context of bioprocesses, real-time measurements of the metabolites and cell concentrations in the cell culture medium are key elements to enable the prediction of cell phenotype with respect to operating conditions (e.g., by using constraint-based modeling approaches)^17^. Unfortunately, some of these experimental measurements cannot currently be achieved online in situ and, doing so, limit our capacity to have a live snapshot of the cell metabolism during the culture process. However, continuous improvements in analytical technologies and the emergence of the single-cell experiments open new perspectives for gaining insights into cell metabolism.

Spectroscopic methods (e.g., NIR, FTIR, mid-IR, Raman, Fluorescence) have attracted an increasing interest for their potential capacity of simultaneous in situ measurement of nutrients, metabolites and cell concentrations. These techniques have been successfully integrated into real-time pharmaceutical manufacturing to provide a “molecular fingerprint” of samples enabling in-process correction needed to ensure the acquisition of a specific quality product^18,19^. However, the capacity to systematically extract accurate quantitative data is, for the moment, limited to a handful number of metabolites (typically, glucose, lactate, glutamine, glutamate, and ammonium). We refer the reader to the publications of Li et al.^20^ and Ryder^21^ for detailed comparison of the performance and limitations of existing spectroscopic methods. In this context, chemometric modeling has been a state-of-art technique to effectively extract the maximum of information from spectroscopic spectra. It is worth to highlight that spectroscopic sensors can in this sense be embedded in the concept of soft sensor^22^ as they are used to compensate the lack of specific measurement by reconstructing the missing signals based on available measurements using data analysis models^23^. This type of hybrid approach will therefore continue to greatly benefit from the flourishing evolution of artificial intelligence tools and certainly allows to overcome the current limitations of spectroscopic monitoring methods^24^.

Parallelly, the rise of online single‐cell probing opens the door for the specific investigation of cell metabolism and bioenergetics at a definition level never captured before^25^. These “single-cell approaches” in combination with adequate analytical methods could help unravel the impact of operating conditions on the cellular physiology. Specifically, the new generation of high-throughput-omics technologies allow to envision in a close future a near real-time measurements of cell transcriptome and proteome^26–28^. Interestingly, very few examples of interfacing flow cytometry with a bioreactor and its use for effective bioreactor control can be found in the literature^29^.

## Complexity of metabolic networks: reduce to better predict

A genome-scale metabolic model is a network connecting all metabolic reactions that can occur in a specific organism with their associated metabolites, proteins and genes. While these models have been proven to be powerful tools for in silico simulation of cell metabolism^12^, the complexity of these large networks also hinders their utility in various practical applications. Actually, metabolic networks involving, most of the time, more reactions than metabolites, the associated system presents, generally, a multitude of solutions (i.e., underdetermined system). Therefore, metabolic network structures are also too complex to be handled for the development of utilitarian tools (e.g., optimization and control of a bioprocess based on cell phenotype prediction).

Different approaches have been proposed for tailoring these metabolic networks based on a priori knowledge and/or available experimental data. For example, algorithms like the one presented in Erdrich et al.^30^ or Ataman et al.^31^ allow an easy-to-implement systematic reduction of genome-scale networks into core models. However, these methods rely on the definition by the user of the part of the model that should be “protected” during the reduction (i.e., specific metabolites, reactions, phenotypes that need to be conserved) which is far from easy task. On the other hand, numerous algorithms have been developed to tailor genome-scale metabolic networks by integrating diverse types of -omics data, including transcriptomics, proteomics and metabolomics^32–35^. Actually, genome-scale models are used to link genes, enzymes, and metabolism through the use of gene-protein-reactions (GPR). Therefore, the knowledge of GPR is used by researchers to explain the metabolic state of a cell, based on the expression of metabolic genes and/or specific proteins^36,37^. The most common “integration algorithms” use gene expression data to recapitulate the metabolism of an organism under a specific condition (e.g., specific cell-line or operating conditions) by only extracting the subset of active reactions from the genome-scale model. These methods have been proven to be valuable for enhancing the accuracy of model-predicted growth rates and gene essentiality^37^. However, since currently no quantitative description of the GPR relationship exists in genome-scale metabolic models, the integration of gene expression data requires the use of strong assumptions to link the GPR expression and the metabolic reaction activity, which could lead to an oversimplification of the complex relation existing between fluxes, enzymes and genes^36,38^.

Finally, it is important to highlight that these “network tailoring” approaches generally do not completely leverage the problem of system underdetermination related to network complexity. Therefore, the choice of adequate strategy to solve the system (e.g., linear optimization techniques such as Flux Balance Analysis^39^) will always be required to achieve an instantaneous picture of the flux distributions in the cell. A recent research in this field presented a new approach using biochemical thermodynamic constraints (i.e., Gibbs energy) to shape the solution space of potential flux distributions^40^. The promising results obtained in this study pave a new way towards the understanding of the mechanisms that governs the definition of metabolic pathway usage across organisms and conditions. Further development of these computational methods will be crucial to obtain a holistic and integrated picture of the cell metabolism in function of specific environmental conditions while considering uncertainty associated with experiments, measurements and modeling.

## Modeling with hybrid approaches: combine to effectively implement

The application of artificial intelligence and machine learning in bioprocess engineering and systems biology has seen a significant increase in the last years thanks to the research advances in these fields^41–43^ and their successful applications for e.g. in tumor detection^44^. In particular, advances in image recognition enable the automation of so far manual tasks, such as the possibility to automate the counting of cell colonies grown on petri-dishes, using digital imaging^45^. Surprisingly, the practical implementation of these hybrid approaches combining machine learning and the extended knowledge available about biological systems is still in its infancy in bioindustries.

Considering the availability of this prior knowledge, hybrid modeling and artificial intelligence approaches have the potential to significantly reduce the amount of experiments that need to be executed, while potentially increasing the region in which the model can reliably generate predictions. Actually, hybrid models seek to complement what is mechanistically known (e.g., about the metabolism and kinetics) with data-driven methods to describe the unknown parts. This type of models provides an attractive method for modeling biochemical processes as much of the underlying complexity can be lumped in the data-driven part. Therefore, these approaches can be used to effectively establish the link between metabolism and operating conditions in a way that can be used to implement control and optimization strategies.

**Fig. 1:**
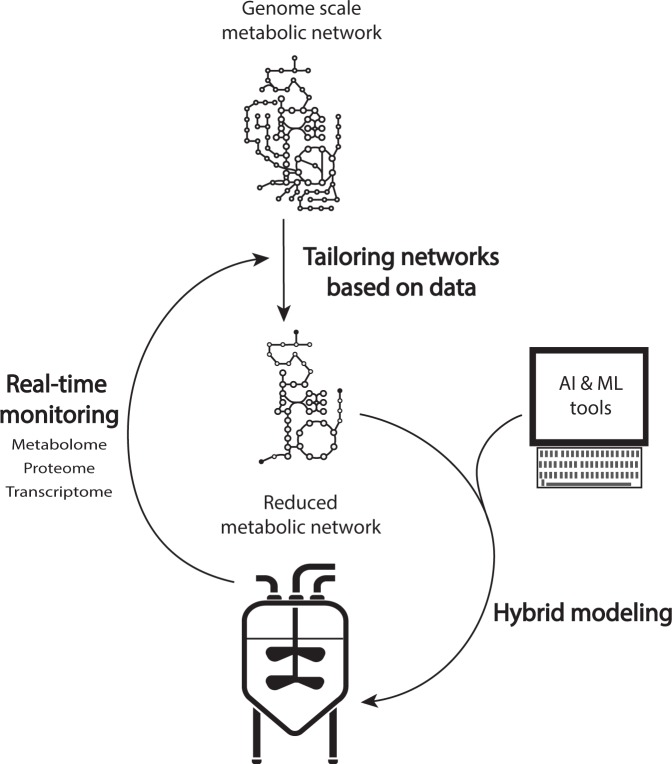
From experimental data to bioprocess improvement. Systematic workflow using data extracted from real-time monitoring to tailor genome-scale biological networks to core metabolic models that can be combined with artificial intelligence and machine learning tools for an effective implementation of control and optimization strategies.

Despite their benefits and their first applications stemming from 1992^46^, the first application of hybrid modeling in systems biology only arose in 2010^47^ and few have been reported ever since^48–53^. One reason might be the absence of dedicated software that would allow for a straightforward development of these type of models. Another reason might be the broad interdisciplinarity of competences required to implement these approaches (i.e., knowledge from engineering fields and data sciences that need ideally to be complemented with a biological background). Finally, there is still several major open questions that need to be investigated to allow a better combined usage of hybrid modeling and metabolic modeling tools^54^. From the effective generation of sufficiently informative experimental data to the joint parameter identification of the mechanistic and data-driven parts of the model, the assessment of these gaps will be critical for the development of a systematic workflow for integrating knowledge from systems biology into model structures enabling a next-generation technology for the life-sciences and biotechnology sectors, besides others (Fig. 1).

## Bridging the gaps towards effective continuous processing

Process intensification through continuous processing has been adopted for decades in industries such as chemical and petrochemical. In the context of biopharmaceutical production, the move from batch to continuous or semi-continuous mode of production has been slowed down mainly due to the more conservative approach of this industry resulting from the stringent quality/regulatory requirements. Therefore, innovation in biotechnology has become more product- than process-centered^55^. While these old drivers of bioprocess development are still valid, new emerging challenges are forcing the change towards extended manufacturing flexibility and transferability. The commitment to deliver on them includes process development timeline decrease, productivity increase and product quality modulation (e.g., for biosimilarity).

The proper implementation of continuous manufacturing requires a thorough understanding of the process to achieve and maintain steady-state conditions (i.e., the causalities between physiological state, bioprocess parameters, productivity and product quality attributes). Furthermore, the in-process quality should be evaluated and ensured by the real-time analysis of process data. To this end, in-line process monitoring system linked to robust model-based control will be crucial technologies to support this real-time release testing^56,57^.

Despite their advantages, the potential of metabolic modeling tools is still not fully exploited to address these challenges in bioprocess development and improvement. This is mainly due to the upfront time and effort investment required to implement these approaches and gain in process understanding as critical gaps remain to set these methods up in clear workflows. Empirical process development techniques will continue to play an important role in addressing these challenges. However, systems biology-driven approaches are likely to be more impactful to provide insights into the cellular responses to process conditions changes^2,14^.

Finally, the research topics, highlighted in this review, in the field of monitoring, network science and hybrid modeling, will also be cutting-edge technologies to improve new emergent biopharmaceutical production platforms such as cell-free systems. Actually, cell-free protein synthesis has been emerging as a flexible and powerful platform to address challenges in biomanufacturing by overcoming inherent limitations related to the use of living cells. Cell-free systems offer the ability to design metabolic pathways towards the production of desired products but also to synthesize complex proteins with unnatural amino acids and to buildup artificial cells. Despite these promising features, challenges remain such as the proper control of post-translational modifications and the expansion of the genetic code for unnatural amino acids incorporation. To address these challenges, targeted gene editing and addition of adequate exogenous substances to control reaction conditions will be needed to optimally regulate the transcription and translation^58,59^. In this context, computational tools will be invaluable to systematically identify system limitations and areas of improvement for production efficiency, as demonstrated in the work of Vilkhovoy et al.^60^.

## Discussion

We highlighted gaps in our knowledge and technical limitations restraining the effective application of metabolic modeling tools throughout the lifecycle of a biopharmaceutical process. Specifically, advances in real-time monitoring of bioprocesses, biological network modeling and their combination with data-driven approaches will be the key drivers to lower the time and cost associated with the development of new drugs^4^. Taken together, these technologies could facilitate the switch to continuous processing in biopharmaceutical industry but also foresee needs for improvements in emergent biomanufacturing platforms such as cell-free systems.

## Supplementary information

Reporting Sum
